# From hospital discharge to long-term care: Unmet rehabilitation needs in cauda equina syndrome patients from a national UK cohort

**DOI:** 10.1016/j.wnsx.2025.100485

**Published:** 2025-07

**Authors:** Holly Roy, Krithika Anil, Jack Read, Marcus J. Drake, Ingrid Hoeritzauer, Julie Woodfield, UCES Collaborators, British Neurosurgical Trainee Research Collaborative

**Affiliations:** a Peninsula Medical School, University of Plymouth and University Hospitals NHS Trust, Plymouth, UK; b School of Health Professions, Faculty of Health, University of Plymouth, Plymouth, UK; c Brain Research and Imaging Centre, University of Plymouth, Plymouth, UK; d University of Exeter Medical School, College of Medicine and Health, Exeter, UK; e Faculty of Medicine, Department of Surgery & Cancer, Imperial College, London, UK; f Royal Infirmary of Edinburgh, NHS Lothian and Centre for Clinical Brain Sciences, University of Edinburgh, UK; g Centre for Clinical Brain Sciences, University of Edinburgh, UK

**Keywords:** Cauda equina syndrome, Spinal cord injury, Postoperative care, Rehabilitation, Nervous system diseases

## Abstract

**Objectives:**

Although long term disability may be a consequence of cauda equina syndrome (CES), the evidence base for the effect of rehabilitation or different rehabilitation strategies is lacking. Our aim was to understand long term neurological deficits (understood as rehabilitation needs) and current rehabilitation provision for patients with CES.

**Methods:**

We retrospectively analysed data from a large UK wide cohort of CES patients presenting between 1st June 2018 and 31st May 2019. Rehabilitation referrals and attendance were described, and symptoms at discharge and one year were identified as potential targets for rehabilitation.

**Results:**

Physiotherapy was the most common inpatient rehabilitation service accessed following surgery for CES (572/610, 94%). Few patients were referred to specialist spinal rehabilitation services at discharge (49/608, 8%). At one year follow up there were high rates of residual symptoms (motor (66%), bladder (20%), bowel (17%), and sexual dysfunction (13%)). There was a significantly higher level of ongoing bladder dysfunction in females (27%) compared with males (11%) despite similar levels at presentation (females 84% vs males 82%).

**Conclusion:**

Referral to specialist spinal rehabilitation following CES surgery is not routine in the UK but a notable proportion of patients have ongoing symptoms at one year following surgical decompression. Prospective studies of rehabilitation strategies following surgery for CES are needed to guide treatment decisions and optimise post-surgical outcomes.

## 1 Introduction

Cauda equina syndrome (CES) arises from compression of the nerve roots which lie in the spinal canal below the termination of the spinal cord.^1^ These are a mixture of sensory, motor and autonomic nerves which innervate the lower limbs and pelvic viscera. Compression of the cauda equina by a prolapsed intervertebral disc, haematoma, infection, tumour or following trauma produces a pre-ganglionic nerve injury, which can result in bowel, bladder, and sexual dysfunction, paralysis or weakness, sensory loss or paraesthesia, and pain^2^. Post-ganglionic peripheral nerves have recognised regenerative capacity,^3^ while the spinal cord and brain do not regenerate effectively, in part due to inhibitory properties of central nervous system (CNS) myelin as opposed to peripheral nervous system (PNS) myelin 4. Less is known about the regenerative properties of the cauda equina, nevertheless, permanent deficits can occur in the context of CES and studies report significant morbidity several years after surgical decompression for CES, encompassing domains including pain, mental health, bowel, bladder, sexual function and ability to return to employment.^5^, ^6^, ^7^, ^8^, ^9^

While the ongoing debate^10^ about timing of surgery has been a key focus of research in the field of CES, less attention has been paid to determining whether post-operative rehabilitation interventions may enhance neurological recovery following successful surgical decompression. Early multi-disciplinary intense rehabilitation has been shown to maximize neurological recovery in traumatic spinal cord injury^4^ and stroke,^11^ and strategies such as pharmacological treatments and neuromodulation may improve outcomes.^12^ The recent National Health Service (NHS) Getting It Right First Time (GIRFT) national suspected CES pathway advises physiotherapy and splints as needed as an inpatient, along with assessment of requirements for ongoing support in the areas of pain, psychology, sexual health, bladder and bowel health, and if symptoms are present at 6–8 weeks post-operatively, referral to the spinal cord injury team.^13^ However, evidence for the effect of rehabilitation after CES is scarce, with one study of inpatient rehabilitation for CES due to spinal stenosis showing an improvement on the Functional Independence Measure (FIM) with inpatient rehabilitation but without a control group for comparison.^14^ A cohort study of 214 patients with CES due to trauma showed an improvement in motor function, FIM and spinal cord independence measure (SCIM) following inpatient rehabilitation, and better outcomes when rehabilitation began in a shorter time period following injury.^15^ However, this study also did not have a control group. A scoping review did not identify any cohort studies of the effect of rehabilitation for those with CES due to disc disease.^16^

Given the debilitating effects of CES, understanding how to optimise post-decompression recovery is an important goal which has not previously been studied. The aim of this study was to explore residual symptomatology in a large prospective, contemporary cohort as a marker for rehabilitation needs in a UK cohort, and describe current rehabilitation practice following CES to identify any mismatch in needs and provision.

## 2 Methods

Data was obtained from a multi-centre observational cohort study of patients with CES in the UK presenting between 1st June 2018 and 31st May 2019. Ethical approval for that study was given by the South East Scotland Research Ethics Committee 02 (18/SS/0047) in April 2018. CES was defined as altered saddle sensation, bladder dysfunction, bowel dysfunction, sexual dysfunction or bilateral sciatica, with associated radiological cauda equina compression. Clinician reported data for rehabilitation services used as an inpatient and for referrals made at discharge were extracted. Patient-reported data for ongoing symptoms and rehabilitation services used as an outpatient up to one year following decompressive surgery were also extracted. Rehabilitation services were grouped as physiotherapy, occupational therapy, continence (including urology, catheter services or clinics, bowel incontinence services, gastroenterology, or other descriptions of services related to bladder or bowel dysfunction), spinal rehabilitation (including any inpatient or outpatient contact with designated specialist spinal rehabilitation services) and other, which could be entered in free text boxes or selected from options such as pain services, neurology, psychological services. Rehabilitation and recovery data were compared between males and females.

### 2.1 Data analysis

Patient demographic information, ongoing symptoms and referral to or use of rehabilitation services or other healthcare services were reported. A Fisher's exact test was used to compare symptoms between groups with a significance level of 0.05.

## 3 Results

### 3.1 Patient characteristics

There were 610 patients with data available regarding inpatient therapy input following admission and surgery for CES (281 male, 329 female; median age 42 years (IQR 33–53 years)). 280 patients (115 male (41 %), 165 female (59 %); median age 43 years (IQR 35–55 years)) provided patient reported information about symptoms at one year and also provided information about therapy specialties seen as an outpatient after discharge (46 % of the original cohort).

### 3.2 Reported symptoms pre-operatively and at 1 year follow up

At initial presentation, 367/610 patients (60 %) reported leg weakness, 507/610 (83.1 %) reported bladder dysfunction, 240/610 (39.3 %) reported bowel dysfunction, and 111/610 (18.2 %) reported sexual dysfunction. 188/610 (30.8 %) had a catheter pre-operatively.

At 1 year follow up, 185/280 (66 %) reported leg weakness, 120/280 (42.9 %) reported bladder dysfunction, 101/280 (36.1 %) reported bowel dysfunction and 80/280 (28.6 %) reported sexual dysfunction. 33/280 (11.8 %) had a catheter 1 year post-operatively.

### 3.3 Rehabilitation and multidisciplinary team input

**Fig. 1:**
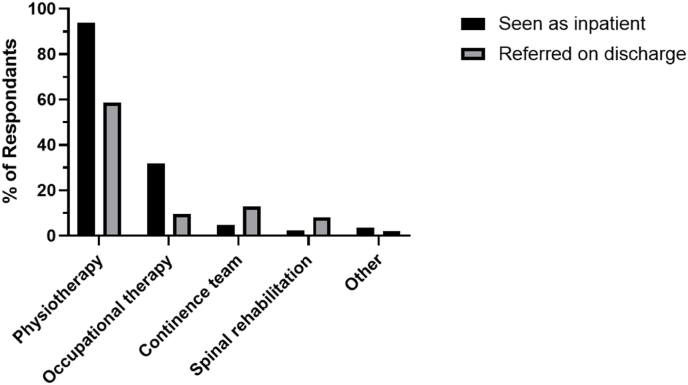
Referrals to various rehabilitation teams as an inpatient or following discharge.

Of the 610 patients with information available about inpatient rehabilitation, 572/610 (93.8 %) received physiotherapy input, 195/610 (32 %) had occupational therapy input, 29/610 (4.8 %) had continence team input and 15/610 (2.5 %) were referred to spinal rehabilitation. Of 608 patients with information about referrals made on discharge, 357/608 (58.7 %) were referred to physiotherapy on discharge from hospital, 58/608 (9.5 %) were referred to occupational therapy, 79/608 (13 %) were referred to the continence team, and 49/608 (8.1 %) were referred to specialist rehabilitation services (Fig. 1).

A total of 280 patients provided data about outpatient specialties seen after discharge. Overall, 133/280 patients (47.5 %) had physiotherapy as an outpatient, 33/280 patients (11.8 %) had occupational therapy, 54/280 (19.3 %) had continence team input (14.8 % of male respondents and 22.4 % of female respondents), 11/280 (3.9 %) had rehabilitation input, 144/280 (51.4 %) saw a GP and 44/280 (15.7 %) saw another specialist from an allied medical specialty or an alternative medical therapy as a self-referred service (chiropractor, osteopath, neurology, psychology, psychiatry, acupuncture, healer, hypnotist, massage therapist, hydrotherapist, counsellor, rheumatology, orthotics, pain specialist, vascular surgeon, hip orthopaedic surgeon, dermatologist).

76/610 patients (12.5 %) required a catheter at discharge (36 male, 40 female; median age 42 (34–55); data does not include information on whether the catheter was indwelling or suprapubic), and of these, 73 (96 %) received physiotherapy, 41 (53.9 %) occupational therapy, 23 (30.2 %) continence team support and 7 (9.2 %) spinal rehabilitation team input as an inpatient. 33/280 (11.8 %) had a catheter 1 year post-operatively, (10 male, 23 female; median age 43 (34–52)). Of the catheterised patients only 7/33 (21.2 %) received spinal rehabilitation input and 23/33 (69.7 %) were followed up by a local continence team.

### 3.4 Differences in outcomes between male and female patients

**Fig. 2:**
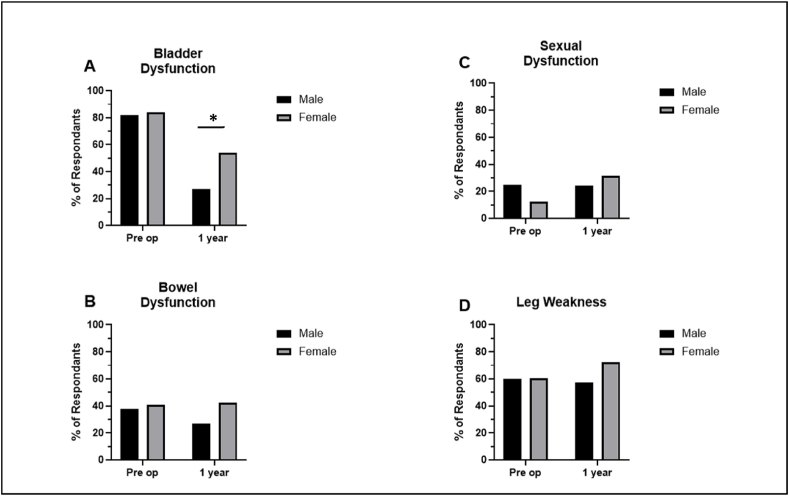
Proportion of patients reporting bladder (A), bowel (B), sexual (C) and motor symptoms (D) pre and post operatively.

We compared patient-reported symptoms at admission and 1 year post-operatively for leg weakness, bowel dysfunction and bladder dysfunction between male and female patients (Fig. 2). The proportion of male and female patients reporting leg weakness (168/281 (59.8 %) male, 199/329 (60.5 %) female), bowel dysfunction (106/281 (37.7 %) male, 134/329 (40.7 %) female) and bladder dysfunction (230/281 (81.9 %) male, 277/329 (84.2 %) female), at the time of admission with CES was similar. Pre-operatively, there was a higher proportion of male patients reporting sexual dysfunction (70/281 (25 %)) than female patients (41/329 (12.5 %). At one year post operatively, the proportion of male and female patients reporting leg weakness was 66/115 (57.4 %) male, 119/165 (72.1 %) female; bowel dysfunction 31/115 (27 %) male, 70/165 (42.4 %) female; bladder dysfunction 31/115 (27 %) male, 89/165 (53.9 %) female; sexual dysfunction 28/115 (24.3 %) male, 52/165 (31.5 %) female. There was no significant difference in recovery of leg weakness or bowel function between the male and female patients over a year, although there was a non-significant tendency for female patients to report more ongoing symptoms in these domains. There was a significant effect of sex (male/female) on the recovery of bladder symptoms at 1 year with 54 % of females and only 27 % of males reporting bladder symptoms at 1 year despite a similar proportion with bladder symptoms pre-operatively (Fisher exact test, p < 0.05). The proportion of male and female patients accessing continence services reflected the higher proportion of female patients with urinary symptoms (17/115 (14.8 %) male and 37/165 (22.4 %) female reported contact with continence services). However, only around half of all male and female patients with urinary symptoms at 1 year reported outpatient contact with continence services.

## 4 Discussion

We analysed presenting and long-term symptoms in a contemporary cohort of patients with cauda equina syndrome and evaluated the rehabilitation options available to the cohort. Overall our study found a mismatch between presence of symptoms and use of rehabilitation services. Referrals to dedicated spinal rehabilitation services were low in this patient cohort , despite over 60 % of patients reporting ongoing symptoms, and a number of patients having multiple symptoms, for example leg weakness and bowel/bladder symptoms. We showed that physiotherapy and occupational therapy were the most common therapies accessed, with 94 % of patients receiving inpatient physiotherapy but only 48 % receiving outpatient physiotherapy despite 66 % reporting leg weakness. Sixteen percent of patients accessed services other than physiotherapy, continence, and rehabilitation. These included alternative and allied therapies such as chiropractor, osteopath, acupuncture, healer, hypnotist, massage therapist, and hydrotherapists. This further suggests that patients are actively seeking out rehabilitation and pain management due to ongoing symptom control requirements not being met with rehabilitation services. We also found that there was a drop-off in utilisation of certain therapy types in the community, with less physiotherapy and occupational therapy but an increase in utilisation of continence services and access to GP. This probably reflects the differences in access and organisation of services between inpatient and outpatient settings.

Our analysis also shows that long-term symptoms are a common feature of CES, with 66 % experiencing permanent motor deficits and 11 % requiring a long-term catheter at one year. This highlights the importance of long term support for this patient group as well as focussed research around interventions, apart from timely surgical decompression, that could enhance long-term outcomes. NHS England/GIRFT have recently published a national pathway for CES, which includes referral to spinal cord injury services for all CES patients with residual symptoms following surgery.^13^ This may improve access to specialist spinal rehabilitation services nationally for CES patients in England by increasing awareness of the need for this pathway. However, no planned expansion of services for this group has been publicised so access and capacity for additional groups may be limited or cause delay in access. There is also no evidence for the relative benefits of locally delivered community rehabilitation, centralised specialist spinal rehabilitation, or any rehabilitation services in CES due to disc disease, so the effect of rehabilitation on outcome is unknown.

We identified a higher rate of persistent bladder symptoms at 1 year amongst female compared with male patients (53.9 % female compared with 27 % male), despite a similar proportion of male and female patients describing bladder symptoms on presentation. This is in line with previous research which suggests that female sex may be associated with worse bladder symptoms following CES.^17^^,^^16^ Podnar.^18^^,^^17^ studied a series of 65 patients with chronic cauda equina lesions and found that urinary incontinence was more common in women than men (74 % of women and 54 % of men with chronic cauda equina lesions reported urinary incontinence), and that the severity of incontinence was also greater in women. The reasons for this are not yet known and may reflect underlying differences in the innervation of the bladder in women. For example, animal studies have shown sexual dimorphism with regard neuronal innervation of the external urethral sphincter.^19^ In males, the bladder neck is innervated by the sympathetic nervous system which should not be affected in CES and provides some protection in the context of external urethral sphincter denervation.This is different to females in whom the bladder neck is predominantly innervated by parasympathetic cholinergic neurons which would be implicated in a cauda equina lesion.^20^ Alternatively, the greater significance of the pelvic floor for the maintenance of female compared with male continence may play a role. Innervation of the pelvic floor muscles, pubococcygeus, puborectalis and iliococcygeus, is from the nerve to levator ani (S4, part of pudendal plexus) and the pudendal nerve (S2-4), all of which would be potentially compromised by central compression of the cauda equina.^21^ The excess urinary incontinence in females may also be influenced by a higher baseline rate of urinary incontinence symptoms in the population in females compared with males.^22^^,^^18^ Our data clearly indicates a strong clinical need to carry out further prospective research to investigate and characterise long term urinary symptoms following CES and to develop a stronger evidence base around differences in rehabilitation needs for male and female patients, including investigation of the role for targeted pelvic floor rehabilitation.

The clinical data available on CES symptoms at 1 year was patient reported only, and future studies are required to phenotype established CES in greater depth, including clinical examination and documentation of other important symptoms including perineal numbness and sexual function. Novel interventions for pain and incontinence after CES including sacral nerve stimulation and spinal cord stimulation have been described at case report level.^23^^,^^24^^,^and warrant further investigation, in larger patient groups for those with ongoing symptoms. Furthermore, early non-invasive neuromodulation should also be considered, as a lower cost intervention which could potentially be accessed at an earlier stage following the identification of chronic symptoms after CES.^25^^,^ Prospective trials are urgently needed to compare outcomes following both standard rehabilitation and novel interventions more rigorously and establish the conditions for their delivery.

Although this paper used a UK cohort only, it is probable that the findings and rehabilitation needs are applicable to populations in other countries. A multicentre, international approach would be useful to confirm this and to investigate rehabilitation practices and outcomes in different countries.

Detailed questionnaire based or physiological data around patient reported symptoms in the UCES patient cohort were not available. Therefore, it is not possible to better understand the precise nature of the long-term motor, sensory and autonomic symptoms experienced by patients, and this limits our ability to interpret the data. Another significant limitation of this study is that the true proportion of patients with ongoing symptoms is not known, as not all patients consented for completion of 1 year follow up questionnaires and therefore proportions may be higher or lower than those given. Finally, we are only able to speculate on the reasons as to why patients were not referred to rehabilitation services. One reason may be that patients were discharged from hospital and expected to continue to recover and mechanisms were not put in place to recognise failure to progress at a later stage. One way to circumvent this problem would be to refer all patients with any residual deficit to rehabilitation at discharge and if the deficits recover while the patient is waiting to be seen, the appointment can be cancelled.

In conclusion, this assessment of rehabilitation available to patients in a contemporary UK cohort shows that while therapy input including physiotherapy, occupational therapy and continence team input was generally available to patients, the proportions accessing these services were lower than the proportions with ongoing symptoms and patients sought alternative therapies, suggesting that needs are not currently being met. Using this retrospective approach it is not possible to evaluate the impact of rehabilitation on outcomes; a prospective study design such as a cohort study or randomised controlled study is needed to investigate this in greater depth. Long term continence outcomes for female patients lagged behind that of male patients, suggesting a potential difference in rehabilitation needs or rehabilitation access (see above) between the two groups.

## CRediT authorship contribution statement

**Holly Roy:** Writing – review & editing, Writing – original draft, Visualization, Formal analysis, Conceptualization. **Krithika Anil:** Writing – review & editing, Writing – original draft, Investigation. **Jack Read:** Writing – review & editing, Writing – original draft, Visualization, Formal analysis. **Marcus J. Drake:** Writing – review & editing, Writing – original draft, Visualization, Supervision. **Ingrid Hoeritzauer:** Writing – review & editing, Supervision, Investigation, Funding acquisition, Formal analysis, Data curation, Conceptualization. **Julie Woodfield:** Writing – review & editing, Visualization, Supervision, Project administration, Investigation, Funding acquisition, Formal analysis, Data curation, Conceptualization.

## Declaration of competing interest

None of the authors have any conflicts of interests to declare.

## Abbreviations

Cauda equina syndrome: CES
Central nervous system: CNS
National Health Service: NHS
Getting it right first time: GIRFT
Functional independence measure: FIM
