# Supplementary material for: From hospital discharge to long-term care: Unmet rehabilitation needs in cauda equina syndrome patients from a national UK cohort
Source: World Neurosurg X. 2025 Jul;27:None. doi: 10.1016/j.wnsx.2025.100485 (PMC13343397; doi:10.1016/j.wnsx.2025.100485)
Supplement: Multimedia component 1 [file mmc1.docx]

**UCES (Understanding Cauda Equina Syndrome) Collaborators**

| **First Name + Middle Name(s)** | **Last Name** |
| --- | --- |
| Mohamed | Abdelsadg |
| Motaz MS | Abulaila |
| Usman | Ahmed |
| Qasim | Ajmi |
| Rafid | Al-Mahfoudh |
| Chadi | Ali |
| Meriem | Amarouche |
| Amin | Andalib |
| Mohit | Arora |
| Mukul | Arora |
| Mariam | Awan |
| Asfand | Baig Mirza |
| Antony | Bateman |
| Iwan | Bennett |
| Imran | Bhatti |
| Peter | Bodkin |
| Lalasa | Bommireddy |
| George | Bonanos |
| Anouk | Borg |
| Alexandros | Boukas |
| James | Bourne |
| Rachael | Brennan |
| Jennifer | Brown |
| Katie | Brown |
| Oliver | Burton |
| Christopher | Busby |
| Neil | Chiverton |
| Simon | Clark |
| Phillip C | Copley |
| Simon | Cudlip |
| Yan | Cunningham |
| Ronan | Dardis |
| Stacey | Darwish |
| Benjamin | Davies |
| Andreas K | Demetriades |
| Saurabh | Deore |
| Chris | Derham |
| Muhammad | Dherijha |
| Gareth | Dobson |
| James | Duncan |
| Andrew | Durnford |
| Alexander ZE | Durst |
| Edward W | Dyson |
| Niall | Eames |
| Ellie | Edlmann |
| Andrew | Edwards-Bailey |
| Anne | Elserius |
| Becca | Elson |
| Mohammed | Fadelalla |
| Daniel M | Fountain |
| Adrian | Gardner |
| Arnab | Ghosh |
| James R | Gill |
| Stella A | Glasmacher |
| Robin | Gordon |
| Gordan | Grahovac |
| Rebecca | Grenfell |
| Awais | Habeebullah |
| Nikolaos | Haliasos |
| Tim | Hammett |
| Cathal John | Hannan |
| Ciaran Scott | Hill |
| Ingrid | Hoeritzauer |
| David | Holmes |
| Kismet | Hossein-Ibrahim |
| Laura | Hughes |
| Muhammad | Hussain |
| Shakir | Hussain |
| Ramez | Ibrahim |
| Aimun AB | Jamjoom |
| Bethan | John |
| Shabin | Joshi |
| Josephine | Jung |
| Oliver | Kennion |
| Muhammad | Khan |
| Adriana | Klejnotowska |
| Ashwin | Kumaria |
| Roberta | LaCava |
| Simon | Lammy |
| Alistair | Lawrence |
| Matthew | Lea |
| Andraay HC | Leung |
| Ignatius | Liew |
| Weisang | Luo |
| Oscar | MacCormac |
| James | Manfield |
| Richard | Mannion |
| Joseph | Merola |
| Pranav | Mishra |
| Khalid Abubaker | Mohmoud |
| Richard | Moon |
| Rory | Morrison |
| Odhran | Murray |
| Ali | Nader-Sepahi |
| Colin | Nnandi |
| Anand | Pandit |
| Nitin | Patel |
| Anita | Philip |
| Michael TC | Poon |
| Kuskoor Seethram Manjunath | Prasad |
| Savva | Pronin |
| Shyam | Pujara |
| Balaji | Purushothaman |
| Kapil | Rajwani |
| Fahid Tariq | Rasul |
| Holly | Roy |
| Ahmed-Ramadan | Sadek |
| Moritz | Schramm |
| Gabrielle | Scicluna |
| Philip J | Sell |
| Roozbeh | Shafafy |
| Himanshu | Sharma |
| Asim | Sheikh |
| Vinothan | Sivasubramaniam |
| Agbolahan | Sofela |
| George | Spink |
| Nisaharan | Srikandarajah |
| Patrick FX | Statham |
| Stuart | Stokes |
| Euan | Strachan |
| Chrishan | Thakar |
| Gopiga | Thanabalasundaram |
| Paul | Thorpe |
| Christian | Ulbricht |
| Anna | Watts |
| Alison | Whitcher |
| David | White |
| Kathrin | Whitehouse |
| Martin | Wilby |
| Julie | Woodfield |
| Ardalan | Zolnourian |
